# Next-Generation Stroke Biomarkers: Bridging the Gap Between Innovation and Translation

**DOI:** 10.3390/jcm15166249

**Published:** 2026-08-12

**Authors:** Emilia Conti, Marzia Baldereschi, Antonio Di Carlo, Giulia Barbieri, Francesco Saverio Pavone, Anna Maria Gori, Betti Giusti

**Affiliations:** 1 National Institute of Optics, National Research Council, Via Nello Carrara 1, 50019 Sesto Fiorentino, Italy; conti@lens.unifi.it; 2 European Laboratory for Non Linear Spectroscopy (LENS), Via Nello Carrara 1, 50019 Sesto Fiorentino, Italy; pavone@lens.unifi.it; 3 Neuroscience Institute, National Research Council, Via Madonna del Piano 10, 50019 Sesto Fiorentino, Italy; dicarlo@in.cnr.it; 4 Department of Experimental and Clinical Medicine, University of Florence, Largo Brambilla 3, 50134 Firenze, Italy; giulia.barbieri@unifi.it (G.B.); annamaria.gori@unifi.it (A.M.G.); betti.giusti@unifi.it (B.G.); 5 Department of Physics and Astronomy, University of Florence, Via Sansone 1, 50019 Sesto Fiorentino, Italy

**Keywords:** stroke biomarkers, multi-omics, single molecule, multi-array system, stroke clinical practice, stroke preclinical research

## Abstract

The identification of valid biomarkers for ischemic stroke would greatly benefit not only diagnostic and prognostic assessment but also the development of new therapies. The development of omics technologies has made it possible to explore various perspectives of the pathophysiology of ischemic stroke, in particular genomics, transcriptomics, proteomics and metabolomics, and well-known clinical variables, such as hypertension, diabetes, heart diseases, and many others. Omics-based research has produced a mass of data evidencing candidate biomarkers at different stages in the disease process. However, further scientific evidence must be presented and affordable, and rapid detection techniques developed before these technologies can be usefully applied to the clinical setting of stroke. Here, we aim to provide stroke clinicians with an updated overview of the contribution of these emerging technologies in the individuation of diagnostic and prognostic biomarkers and the development of specifically targeted therapies. Cross-fertilization (translation and reverse translation) between preclinical and clinical research is of particular relevance in the identification of stroke biomarkers. We believe it is useful to provide, in parallel, an updated review of omics investigations using stroke rodent models. This review presents a detailed discussion of the state of the art, focusing on the strengths and limitations of the individual technologies and an outline of the promising, most recent results of both clinical and preclinical research in this field related to stroke.

## 1. Introduction

Ischemic stroke (IS) is a leading cause of disability and mortality worldwide. As individuals age, the risk of experiencing a stroke significantly increases, making stroke a major health concern among the elderly population [1]. Clinical care in the acute phase is currently based on neuroimaging and clinical assessment [2]. However, many aspects remain unaddressed or unsolved. There is a need for: (i) a rapid, accurate blood test to identify IS in the pre-hospital phase and to accelerate triage and access to reperfusion treatments in the narrow therapeutic window; (ii) specific biomarkers to predict and possibly counteract futile recanalization that occurs in about half of recanalized patients; and (iii) diagnostic biomarkers for transient ischemic attacks, because the great majority of patients are asymptomatic when examined, and therefore the diagnosis is only anamnestic [3,4]. These challenges reflect the complex and heterogeneous biological nature of IS. Similar clinical features may be caused by distinct pathogenetic mechanisms that circulating biomarkers might identify, thus allowing for rapid clinical decisions. The identification of reliable biomarkers requires approaches capable of individuating the multiple biological processes involved in stroke initiation, progression, and recovery. Omics technologies provide a comprehensive framework for the investigation of molecular alterations at multiple levels and may help overcome some limitations of conventional biomarker discovery strategies. To address the urgent need for reliable and actionable stroke biomarkers a panel of international stroke experts has recently launched a Delphi consensus survey that identified 17 research priorities for stroke biomarker studies [5].

**Figure 1:**
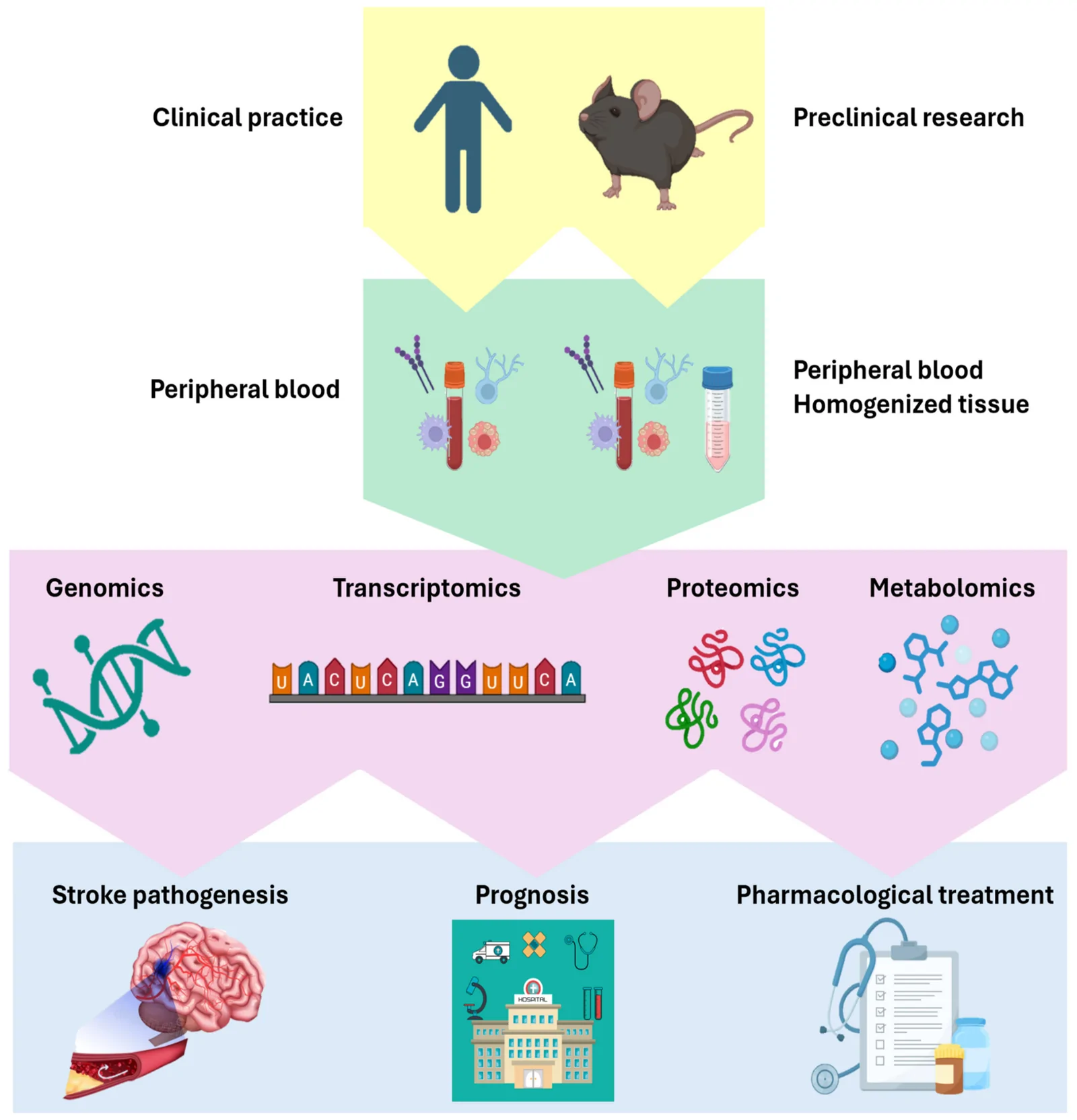
Multi-omics approaches for stroke biomarker identification: from preclinical to clinical research and backwards. Created in BioRender. Pavone, F. (https://BioRender.com/h2saxtv) is licensed under CC BY 4.0.

Current knowledge at the molecular, cellular, and network levels is inadequate for a full understanding of the processes underlying stroke progression. An individual patient outcome is influenced by intrinsic differences in the flow capacity of collateral vessels, degree of chronic ischemic disease, ischemic preconditioning, oxidative stress tolerance, microvascular blood flow regulation, and other still unknown factors. Within this framework, preclinical research may help clarify the neuronal and vascular underpinnings of stroke progression and identify potential treatment targets leading to clinical translation. The main advantage of animal models lies in the possibility of selecting various factors, such as genetic background, sex, and age. On the other hand, stroke models allow for dissecting critical features of stroke pathophysiology by employing in vivo and ex vivo strategies, which is not feasible in clinical practice. This feature, together with the high reproducibility of stroke models, allows for the use of fewer biological replicates while minimizing confounding factors. In this framework, preclinical models, combined with high-throughput omics technologies, can provide the otherwise unattainable mechanistic information at the molecular level underlying functional outcome in stroke patients. The successful translation of omics discoveries into clinical practice relies on the continuous mutual interaction between preclinical and clinical research. On the one hand, experimental models provide a controlled environment for the investigation of pathophysiological disease mechanisms, and they can validate already identified candidate biomarkers in patients. On the other hand, clinical studies are essential to confirm their relevance in the heterogeneous human population. In parallel, clinical cohort studies can guide the development of more representative experimental models and generate new mechanistic hypotheses. This bidirectional framework is essential to accelerate the identification of robust biomarkers and therapeutic targets for IS as it is sketched in Figure 1.

Improvements are needed across the whole stroke care pathway, from reperfusion treatments to long-term rehabilitation, to reduce the medical and societal burden of stroke. We consider continuous cross-fertilization between clinical and preclinical research that is necessary to provide novel solutions to unmet clinical needs.

The aim of this review is to illustrate the emerging technologies that could play a strong role in stroke therapy in the near future. Regarding stroke, omics technologies are currently relegated to clinical and preclinical research, but they may soon become an important asset in stroke care. Moreover, the development of advanced detection approaches, such as single-molecule assay (SIMOA) and multi-array systems, in addition to the development of the Point of Care Test (POCT), might make it possible to assess biomarkers at bedside, even in the pre-hospital phase, thus contributing to the fundamental aspect of timing in IS care.

We discuss the application of omics technology in experimental and clinical settings, emphasizing its potential role as a bridge between mechanistic discoveries and the development of clinically relevant biomarkers in stroke. The aim is to update stroke clinicians on the state of the art, as well as the strengths and limitations of each type of technology, giving an overview of the most recent and promising results from both clinical and rodent stroke model omics research. In addition, we highlight possible bottlenecks in the clinical translation of research findings.

## 2. Integrated Omics (Genomics, Transcriptomics, Proteomics, and Metabolomics)

### 2.1. Genomics

Genetics and genomics could revolutionize stroke care by shifting the focus from reactive treatment to proactive prevention and personalized medicine. Genetic technologies for stroke have been recently reviewed [6]. The study of genomics in stroke has already given promising results related to prevention (risk reduction), the prediction of response to acute-phase treatments (therefore the outcome), and the identification of the different degrees of response to antiplatelet drugs (pharmacogenomics) [7,8]. Apart from rare Mendelian forms, IS must be considered a polygenic and multifactorial disease. Accordingly, recent studies have increasingly used Genome-Wide Association Studies (GWAS), scanning the whole genome to look for genetic variants associated with an increased risk of stroke [8]. Recent large-scale genomic investigations have substantially expanded the catalogue of IS-associated susceptibility loci and improved the biological interpretation of GWAS findings by integrating genetic association data with functional genomic analyses, including the expression of quantitative trait loci (eQTL) and protein quantitative trait loci (pQTL), Mendelian randomization, and single-cell transcriptomic analyses. These approaches have allowed for the identification of new candidate genes regarding vascular biology, inflammation, lipid metabolism, iron homeostasis, endothelial dysfunction, and neuronal injury. In all likelihood, they will lead to the identification of new therapeutic targets and biomarkers for precision medicine in IS [9,10]. As reported in Table 1, several genetic loci have been identified with IS diagnosis, complications and prognosis. The identification of low metabolizers of the antiplatelet clopidogrel is of particular relevance: CYP2C19, encoding an antiplatelet-metabolizing enzyme, has proven to be of extreme interest for primary and secondary stroke-prevention therapies. CYP2C19’s loss-of-function alleles have been proven to be associated with higher risk of recurrent IS in patients on clopidogrel [11,12]. Poor metabolizers turned out to have lower clopidogrel activation and a nearly twofold risk of recurrent stroke than normal metabolizers [13]. These findings suggest that genotyping stroke patients for CYP2C19 is worthwhile, because poor metabolizers can be treated with other P2Y12 inhibitors, such as ticagrelor, which has been proven to be effective in reducing the risk of stroke recurrence in CYP2C19 LoF carriers [14]. It is well known that the frequency of the polymorphism and the related phenotype is closely linked to ethnicity. Although most of the early studies were conducted on populations of European ancestry, there is now a considerable body of evidence also on populations of different ancestries. In European ancestry populations, the frequency of the loss-of-function allele is about 15%, while it is 30% in South Asians and 60% in native Oceanians [15].

#### 2.1.1. Genetic Risk Score

A genetic risk score (GRS) summarizes the small effects of several Single-Nucleotide Polymorphisms, previously identified by GWAS, to help improve risk prediction and stratification beyond traditional risk factors and to facilitate personalized prevention strategies [16]. It is well known that the stroke phenotype is highly heterogeneous, and this limits its genomic risk prediction [17]. Recent studies have adopted the construction of the increasingly powerful GRS based on summary statistics of multiple sets of GWAS to overcome their single limited predictivity and to achieve added value in combination with established clinical risk factors [18]. In particular, recent promising findings relate to atrial fibrillation (AF) and stroke and are summarized in Table 1. When a large-scale GRS was incorporated into the CHA2DS2-VAS score, there was a net improvement in levels of stroke risk classification for 2.3% of AF patients [19]. The clinical implication is that if several patients are moved to a different stroke risk profile, this refinement might be particularly useful for “gray area” patients (i.e., those with low or intermediate risk of stroke) who may hide a significant stroke genetic susceptibility [19]. Moreover, the specific GRS performed better than the traditional clinical score (HAS-BLED) [20] in stratifying the risk of intracerebral hemorrhage in patients on anticoagulants [21]. We note that the highest level of predictivity is achieved when GRS data are added to clinical factors. Finally, a reliable estimate of stroke risk in AF patients remains challenging [19]. Recent evidence further supports the role of polygenic risk scores as complementary tools for identifying individuals at increased genetic susceptibility, refining risk stratification and facilitating future implementation of precision prevention strategies in ischemic stroke [9,10].

#### 2.1.2. Preclinical Studies

Regarding preclinical research, in a recent study on mice, Wang and colleagues explored the biological significance of candidate IS-associated genes [9]. Existing mouse knockout models from the Mouse Genome Informatics (MGI) database were leveraged to establish functional correlations [22]. Of the seven prioritized genes, only HSD17B12, ERAP2, and SFXN4 showed strong experimental support. Loss of HSD17B12 led to disruptions in fatty acid metabolism, highlighting a mechanistic link between lipid metabolic imbalance and cerebrovascular pathology. Similarly, ERAP2 and SFXN4 were implicated in immune modulation and iron homeostasis, two processes known to contribute to stroke onset and progression. Although knockout models are not yet available for other genes, in particular C15orf40, CPNE1, LYRM9, and SNX32, existing annotations suggest potential involvement in neuronal development, cell-cycle control, and mitochondrial activity, indicating their possible roles in neuronal vulnerability during ischemic injury. Notably, drug-potential analyses indicate that three of these genes (CPNE1, HSD17B12, and ERAP2) encode proteins already targeted by approved or clinically tested compounds, underscoring their translational relevance and potential as therapeutic entry points in stroke precision medicine [23,24,25].

### 2.2. Transcriptomics

Gene activity can be investigated through transcriptomic analyses, which encompass bulk RNA sequencing, single-cell RNA sequencing, spatial transcriptomics and extracellular vesicle RNA profiling, providing complementary information on gene regulation in tissue and cells.

Transcriptomic studies on peripheral blood have been extensively and recently reviewed [26] and so have the studies on clot transcriptomics—obtained from endovascular disobstruction interventions [27].

In addition to messenger RNA (mRNA), the transcriptome includes non-coding RNAs (ncRNAs), which do not encode proteins, but they function as regulatory molecules modulating gene expression at the post-transcriptional level [28]. These ncRNAs include microRNAs (miRNAs), lncRNAs, and circular RNAs (circRNAs). miRNAs are small (~21 nucleotides) tissue- and time-specific regulators expressed across human tissues that control gene expression by inducing silencing or degradation of target mRNAs.

Notably, miRNAs released from ischemic brain tissue can cross the blood–brain barrier (BBB) within extracellular vesicles or exosomes and remain stable in biofluids such as plasma and serum, making them promising biomarkers of disease status and prognosis, enabling early identification of patients at risk for poor recovery and possibly informing rehabilitation strategies [29,30]. A specific miRNA signature might possibly support the differential diagnosis. Promising findings may arise from clot transcriptome analysis, which was found to be effective in differentiating the occlusion of cardioembolic origin, rich in red blood cells and fibrin, from that caused by large-vessel atherosclerosis, where platelet and immune activations are predominant, thus assisting in stroke subtyping [31]. In a recent metanalysis of 44 studies (4302 IS patients and 3725 healthy controls) a specific panel of lncRNA showed moderate accuracy (79% sensitivity and 88% specificity) in identifying IS [32]. Cell-free nucleic acids (both DNA and RNA) may lead to the identification of upstream biomarkers for stroke diagnosis, even in pre-hospital settings: testing is minimally invasive and potentially possible by point-of-care testing. A recent systematic review and metanalysis [33] did not provide any conclusive data. Recent single-cell RNA sequencing (scRNA-seq) and spatial transcriptomics have identified cell-type-specific transcriptional responses after ischemic stroke, evidencing distinct activation states of the different cell types involved in IS. These novel approaches have furnished several interesting findings on previously unidentified inflammatory and neurovascular pathways involved in ischemic injury and recovery while preserving tissue spatial organization.

The integration of transcriptomic datasets with GWAS findings and other omics layers has improved the prioritization of causal genes and molecular pathways, supporting the development of precision medicine strategies and individualized therapeutic targets [34,35]. Table 1 summarizes the most relevant findings in transcriptomic human IS research.

#### Preclinical Studies

Preclinical studies show a substantial overlap between differentially expressed genes in peripheral blood and brain [36,37,38]. Androvic et al. recently reported that in aged mice after stroke, genes involved in axonal and synaptic maintenance were downregulated, while type I interferon (IFN-I) signaling was upregulated [39]. Recent studies have investigated the neuroprotective role of miRNA expression in the hyper-acute phase after injury. Song et al. [40] investigated miR-140-5p in a mouse model of Middle Cerebral Artery Occlusion (MCAO), demonstrating reduced expression after stroke compared to controls. Using qRT-PCR, they showed that miR-140-5p modulates the TLR4/NF-κB pathway and that its upregulation inhibits neuronal apoptosis, suggesting a potential role in limiting ischemic damage. Similarly, Kolosowska et al. [41] examined miR-669c, a member of the chromosome 2 miRNA cluster induced by harmful stimuli, and its involvement in stroke-related neuroinflammation. Intracerebral overexpression of miR-669c following stroke modulated microglial/macrophage activation by directly targeting MyD88, a key adaptor of TLR and IL-1 signaling. Lentiviral-mediated overexpression of miR-669c in a transient MCAO mouse model suppressed pro-inflammatory responses while promoting alternative microglial/macrophage activation, supporting its neuroprotective role. In another recent study [42] Metha et al. reported that the brain-specific lncRNA Fos downstream transcript (FosDT) is markedly upregulated following focal ischemia in rodents. FosDT deficiency reduces brain injury by limiting inflammation, apoptosis, mitochondrial dysfunction, and oxidative stress. Moreover, siRNA-mediated FosDT inhibition administered during the hyperacute or acute phase after transient MCAO significantly improved motor recovery and reduced infarct volume. Similarly, Li et al. [43] identified the lncRNA MEG3 as being overexpressed after stroke in MCAO mice; its inhibition decreased lesion volume and cerebral edema while improving neurobehavioral outcomes.

Spatial transcriptomics is emerging as a technology capable of evaluating gene expression in situ and preserving brain architecture, the infarct area in particular, thus bridging the gap between anatomical area and single-cell genomics. The ischemic lesion is not a uniform area of damage but a highly structured tissue environment, with different cellular populations set up into niches defined by unique transcriptional signatures [44]. This technology has the potential for an in-depth study of the ischemic penumbra, i.e., the salvageable tissue, and possibly to identify molecular targets that are able to predict reperfusion injury [45].

### 2.3. Proteomics

During an IS, many proteins (expression of neuroinflammation, BBB leakage, and excitotoxicity) change immediately and then over time in quantity, structure, and function. Proteomics allows for the simultaneous identification and quantification of thousands of proteins and is expected to be considered essential for the knowledge of pathophysiological mechanisms of IS because it allows for an investigation of the huge numbers of protein changes that occur in blood, cerebrospinal fluid, and brain tissue, which hopefully will lead to the identification of clinically relevant biomarkers [46].

Both mass spectrometry (MS)-based proteomics and highly multiplexed targeted proteomics have become powerful approaches for quantifying stroke-related changes in proteins, thanks to advances in instrumentation and bioinformatics. There are currently two different approaches in discovery proteomics: one uses untargeted MS to identify thousands of proteins without a priori hypothesis, because it scans the entire proteome; the other involves targeted multiplex platforms, including Olink^®^ Explore (part of Thermo Fisher Scientific, Waltham, MA, USA) and SomaScan^®^ (SomaLogic Operating Co., Inc., Boulder, CO, USA), to quantify preselected proteins with high sensitivity and excellent reproducibility, even for small quantities, complementing untargeted MS-based discovery proteomics [47].

Studies employing high-resolution MS have identified different expressions of hundreds of proteins after symptom onset that may differentiate ischemic from hemorrhagic insult when neuroimaging is unavailable or delayed [48]. Using the same technology, two-protein panels proved accurate in distinguishing the onset of symptoms within the 4.5 h therapeutic window from those beyond this limit [49].

Parallel efforts yielded a four-protein panel that is strongly related to large-vessel occlusion (LVO) IS, which could possibly assist in diagnosis and treatment decisions. Furthermore, a three-protein panel turned out to be significantly associated with collateral status and proved to be an independent predictor of good outcome [50]. Moreover, a panel of 17 proteins proved to be associated with hemorrhagic transformation [51]. In a population-based large-cohort study, distinct pre-stroke protein profiles identified by the Somascan technique proved capable of distinguishing between embolic and atherosclerotic stroke, suggesting the possible development of personalized stroke-prevention strategies [52]. One recent study demonstrated the accuracy and the feasibility of analyzing the proteome in extracellular vesicles (EVs) isolated from small amounts of human plasma/serum. Because EVs preserve the molecular cargo of their cells of origin, they may provide higher tissue specificity than unfractionated plasma proteomics [53]. Specific proteomic signatures of the EV cargo proved accurate in differentiating healthy controls from LVO IS and from hemorrhagic stroke [54].

Although blood-based proteomics remains the most immediately translatable approach, emerging evidence indicates that extracellular vesicle- and thrombus-derived proteomics may provide complementary biological information, enabling improved stroke subtyping, the prediction of treatment response and the identification of novel therapeutic targets [55,56].

#### Preclinical Studies

Proteomic studies in animal models are crucial for mapping the molecular events underlying acute IS pathophysiological processes and for elucidating possible mechanisms of action of therapeutic interventions. By applying Isobaric Tags for Relative and Absolute Quantitation (iTRAQ)-based proteomics, Datta et al. found an increased expression of many brain-specific proteins (Glial Fibrillar Acidic Protein, GFAP; Ubiquitin C-terminal Hydrolase—L1, UCH-L1; and S100 calcium-binding protein B, S100B) in rat serum [57]. This finding indicates that the hyper-acute opening of the BBB may allow for a leakage of these proteins into the circulation, suggesting a potential role of prognostic biomarkers for predicting therapeutic outcome or disease progression. In other studies, proteomics has been applied to explore molecular mechanisms linked with the development of tissue damage. Zheng’s analysis of rat brain tissue in the acute phase after IS identified 282 proteins (73 upregulated, 209 downregulated) involved mainly in energy liberation, intracellular protein transport, and synaptic plasticity regulation, but they were also identified in neurodegenerative disease-related pathways, including Alzheimer’s Disease and Amyotrophic Lateral Sclerosis, in rat studies [58].

In addition, proteomics has been applied in the assessment of alterations in the chronic phase after stroke. Cortical tissue analysis of MCAO rats from the subacute to chronic phase after injury highlights an altered expression of 1305 proteins within the first 14 days after damage. Moreover, cytoskeleton and synaptic structures, energy metabolism, and inflammatory response were found to be significantly disrupted in the subacute phase after stroke. However, in the long-term phase, there was recovery of the cytoskeleton and the activation of inflammatory pathways, which differed from those activated during the subacute phase [59].

### 2.4. Metabolomics and Lipoproteomics

Metabolomics is uniquely suited to investigate the earliest biochemical changes after stroke onset and to identify clinically relevant biomarkers [46,60,61]. Metabolites are low-molecular-weight molecules derived from lipids, amino acids, carbohydrates and nucleotides; they reflect the downstream products of gene expression, protein activity and environmental influences [46,62,63]. More than 25,000 have been counted in peripheral blood [60]. Owing to their small size, metabolites are able to pass the BBB, so circulating metabolites rapidly mirror cerebral metabolic alterations occurring during acute ischemic stroke. The metabolomic and lipoproteomic approach allows us to investigate the acute phase of stroke, because the natural history of acute IS is linked to metabolic changes that occur in the first minutes and hours. Thanks to the BBB disruption in the early phase of IS [61], metabolomics/lipoproteomics can systematically detect the biochemical profile and its changes over time in peripheral blood samples, providing insights into stroke research [64,65]. The use of this approach is useful for stroke clinicians in the identification of biomarkers for stroke subtyping [65], as well as for predicting reperfusion injury [66]. A recent study [67] suggests the possibility of a “metabolic clock” to identify the onset of stroke and therefore the access to time-dependent treatments: biliverdin and nicotinamide N-oxide (NAMO) combined have been shown able to distinguish time from onset, ≤4.5 h vs. >4.5 h, with substantial accuracy. The advent of mechanical thrombectomy has also enabled direct metabolomic analysis of cerebral thrombi. Comparative metabolomic profiling of thrombi and paired serum samples demonstrated distinct metabolic signatures and lipid enrichment within thrombi, suggesting that thrombus metabolomics may improve stroke etiological classification while providing insights into thrombus biology and the mechanisms underlying treatment resistance [68]. Finally, recent multi-omics reviews suggest that integrating metabolomics with proteomics, transcriptomics and genomics is likely to improve biomarker discovery, patient stratification and precision medicine approaches in IS [46,62,63].

Although no metabolomic biomarker has yet entered routine clinical practice, accumulating evidence indicates that integrated metabolomic and lipidomic profiling may substantially improve stroke diagnosis, etiological classification, treatment selection and outcome prediction, particularly when combined with proteomic and transcriptomic data [62].

Major findings are reported in Table 1.

#### 2.4.1. Preclinical Studies

Animal metabolomic studies outline the progression of stroke, from excitotoxicity to metabolic failure. Although the brain represents only 2% of body mass, it accounts for about 20% of resting metabolism [69]; therefore, arterial occlusion rapidly disrupts energy metabolism, with notable changes in metabolites such as glucose, lactate, creatine/phosphocreatine, and TCA cycle intermediates [70,71,72]. The accumulation of glucose and glycolytic intermediates is a hallmark of ischemia-induced metabolic dysregulation in rodents [73]. Luo et al. [74] applied 5-(diisopropylamino)amylamine derivatization–UHPLC-Q-TOF/MS to cerebrospinal fluid from permanent MCAO rats to investigate these metabolic changes. In addition, post-stroke energy depletion triggers the rapid release and reuptake of glutamate, the main excitatory neurotransmitter in the CNS, thus activating NMDA receptors and causing massive calcium influx from extracellular and intracellular stores. This cascade elevates free radicals and nitric oxide, activates lipases and proteases, and disrupts mitochondrial function, leading to energy imbalance. Kagiyama et al. showed that I-phenylalanine selectively and reversibly suppressed glutamate receptor activity at excitatory synapses in rats and mice, producing a compensatory response to neurotoxic glutamate levels [75]. Post-ischemic inflammation disrupts phospholipid metabolism, altering lipid composition and making brain cells, rich in lipids, particularly vulnerable [76]. Long-term studies in MCAO mice showed persistent changes in lipid metabolism, including increased plasma triglycerides, free fatty acids, and adipokine release [77].

**Table 1:** Summary of the major possible contributions of omics technologies to the study of human stroke by stage of the disease, including application scenario and candidate biomarker.

Ischemic Stroke Stage	Possible Contribution	Promising Application Scenario	Candidate Biomarkers [References]
GENOMICS			
Primary and secondary prevention	Identification of novel causal genes and biological pathways through integrative multi-omics analyses	Precision medicine, therapeutic target discovery and personalized risk prediction	Novel genes identified by integrative GWAS/eQTL/pQTL/TWAS/PWAS analyses (e.g., genes involved in inflammation, endothelial dysfunction, lipid metabolism and iron homeostasis) [10,35]
Primary and secondary prevention	Risk stratification for Large-Artery Atherosclerotic Stroke	Guidance on correct therapy and close monitoring of adherence and its effectiveness	CDKN2A/CDKN2B genes [78,79,80,81], HDAC9 [81] and LPA MMP12 [10]
Primary and secondary prevention	Risk stratification for small vessel disease	Guidance on correct therapy and close monitoring of adherence and its effectiveness	COL4A1/COL4A2 [82] FOXF2, HTRA1, PRDM16, and PMF1 [10]
Primary and secondary prevention	Risk stratification for ischemic stroke	Guidance on correct therapy and close monitoring of adherence and its effectiveness	SORT1 [83] HDAC9, HTRA1, COL4A2, and ABO [10]
Primary and secondary prevention	Screening for atrial fibrillation	Strict monitoring of anticoagulant therapy	PITX2 [84], ZFHX3 [85] and PRRX1 [10]
Primary and secondary prevention	Improving stroke risk stratification in patients with and without atrial fibrillation by genetic risk score	Medication guidance and monitoring	8 genetic loci [19,86,87]
Antiplatelet therapy	Identifying low metabolizer of clopidogrel	Medication guidance	CYP2C19 alleles [11,12,13,14]
Outcome/Prognosis	Prediction of early neurological instability	Supporting treatment decisions	ADAM23 [88]
Outcome/Prognosis	Prediction of 3-month functional outcome	Possible future tool to support prognostication	PPP1R21 [89,90]
TRANSCRIPTOMICS			
Pathophysiology/Diagnosis	Cell-specific molecular mechanisms	Identification of cellular mechanisms and therapeutic targets	Single-cell RNA sequencing (scRNA-seq) signatures [34,35]
Pathophysiology/Diagnosis	Spatial organization of ischemic injury	Identification of neurovascular and inflammatory pathways	Spatial transcriptomic signatures [34,35]
Diagnosis	Early diagnosis	Point-of-care panels	Cell-free DNA and RNA [33]
Diagnosis	Differential diagnosis stroke vs. transient ischemic attack	Guidance for clinical work-up and treatment decisions	Micro RNA23b-3p, 29b-3p and 21-5p [91,92]
Diagnosis	Diagnosis of ischemic stroke	Supporting diagnosis when and where CT is not available	Long non-coding RNAs H19, GAS5, PVT1, TUG1, and MALAT1 [32]
Stroke subtyping	Differential diagnosis of cardioembolic vs. atherosclerotic (clot transcriptomics)	Etiological classification and personalized secondary prevention	PPBP/CXCL7, ITGA2B, GP9, VWF [27,31], and cell-type-specific transcriptomic signatures [34]
Prognosis	Prediction of hemorrhagic transformation before t-PA administration	Customizing t-PA procedure and introducing neuroprotection	A 6-gene profile (SMAD4, INPP5D, VEGI, AREG, MARCH7, and MCFD2) [93]
Prognosis	Prediction of 30-day functional outcome	Supporting prognostication and druggable targets	ATP2B, GRK5, SH3PXD2A, CENPQ, HOXC4, HDAC9, BNC2, PTPN11, PIK3CG, CDK6, and PDE4DIP; TLR2 and TLR4 expression [94,95]
Prognosis	Integrated transcriptomic prediction models	Machine-learning-based prognostic signatures	Integrated mRNA/lncRNA/miRNA signatures [34,35]
PROTEOMICS			
Diagnosis	“Molecular clock” to identify therapeutic window: to distinguish onset before 4.5 h from that after 4.5 h	Estimation of symptom onset when unknown	Protein 4.2 (EPB42) and Phosphatidylethanolamine-binding protein (PEBP1) [49]
Diagnosis	Differentiating ischemic from hemorrhagic insult	Supporting diagnosis when and where CT is not available	Specific proteomic signature [96,97]
Diagnosis	Distinguishing ischemic stroke from stroke mimics	Supporting challenging differential diagnosis	30 proteins [98] and 17 peptides [99]
Diagnosis	Distinguishing minor ischemic stroke and transient ischemic attack from non-vascular conditions	Supporting physicians in challenging differential diagnosis in time-sensitive situations	IGFBP3 (insulin-like growth factor-binding protein-3) and PON3 (serum paraoxonase/lactonase-3) [100,101]
Diagnosis	Identifying large-vessel occlusion ischemic stroke and hemorrhagic stroke versus healthy controls	Supporting diagnosis when and where CT is not available	Proteomic patterns of extracellular vesicles [54,96]
Stroke subtyping	Identifying stroke patients with large-vessel occlusion versus non-stroke controls	Supporting stroke subtyping when imaging is not clear	PPBP, THBS1, LYVE1, and IGF2 [50]
Stroke subtyping/Prognosis	Distinguishing atherothrombotic and cardioembolic strokes, favorable reperfusion and outcome (clot proteomics)	Supporting stroke subtyping and therefore related secondary prevention treatments, as well as prognostication	Thrombus proteomic signatures associated with stroke etiology, thrombolysis responsiveness and clinical outcome [102,103,104]
Prognosis	Identifying good collateral brain blood circulation and predicting good 3-month functional outcome	Supporting evaluation of collateral vessels when imaging is not clear and related prognostication	IGF2, LYVE1, and THBS1 [50]
Prognosis	Prediction of both favorable and unfavorable outcomes	Improving prediction of final outcome beyond traditional clinical features	9 proteins [105]
Prognosis		Biological response to thrombolysis	Prediction of thrombolysis responsiveness and identification of therapeutic targets
METABOLOMICS			
Diagnosis	“Metabolic clock” to identify therapeutic window: to distinguish onset before 4.5 h from that after 4.5 h	Helping physicians estimate time from stroke onset when it is not clear or not reported	Combined biliverdin and nicotinamide N-oxide [67]
Diagnosis	To diagnose acute ischemic stroke vs. control	Supporting diagnosis when and where CT is not available	30 metabolites [106]
Treatment response/Stroke biology	Thrombus metabolic characterization	Understanding thrombus composition and mechanisms of treatment resistance	Thrombus-specific lipid metabolites [68]
Prognosis	Prediction of reperfusion injury	Customizing endovascular procedure and introducing neuroprotection	Methionine, acetate, GlyA, MMP-2, CXCL-10, IL-12 and LDL-5 [66]
Prognosis	Predicting unfavorable outcomes (worst 3-month functional outcome) and symptomatic hemorrhagic transformation with non-response to rt-PA	Customizing recanalization treatments, introducing neuroprotection. Improving prediction of final outcome beyond traditional clinical features	3-hydroxybutyrate, acetone, triglycerides high-density lipoprotein (HDL) and triglycerides, low-density lipoprotein (LDL), cholesterol and phospholipid levels [64]

The investigations are blood-based, unless otherwise specified.

#### 2.4.2. Integromics

Although most of the clinical and preclinical studies discussed above have focused on single-omics approaches, increasing evidence indicates that the integration of multiple omics datasets may better highlight the multifaceted pathophysiology of stroke.

The integration of individual omics investigations combines complementary information from different biological levels, such as genomic variation, gene expression, protein abundance, and metabolic alterations, allowing for the identification of molecular networks rather than only isolated biomarkers [107].

A recent integrative study [9] combining multiple large-scale genomic datasets with transcriptomic and proteomic data has helped to identify candidate genes and possible biological pathways involved in IS.

In another work, Jung et al. have demonstrated the potential of integrative-approach profiles to move beyond single-layer analyses and identify molecular mechanisms underlying ischemic injury [108]. As an example of system-level integration, Jung et al. applied a multi-omics framework combining genomic, transcriptomic, epigenomic regulatory, and proteomic information to investigate IS susceptibility. By integrating GWAS findings with molecular quantitative trait loci data, the study provided a more comprehensive view of how genetic variation may influence molecular regulation and disease risk, illustrating the added value of integromics compared with isolated omics approaches.

These recent studies provide preliminary information regarding the use of integrated omics to develop future preventive or therapeutic strategies. The combinations of genomics and transcriptomics [9] or genomics with transcriptomics and proteomics [108], epigenomics, single-cell sequencing, and advanced machine-learning approaches have led to advancements in the development of stroke precision medicine.

Despite these promising advances, integrative multi-omics approaches in stroke research are still in their early stages. Nevertheless, recent studies have already demonstrated their ability to identify robust biomarker panels, molecular subtypes, regulatory networks, and candidate therapeutic targets, highlighting the potential of systems biology to advance precision stroke medicine [10,63,109]. Future studies integrating larger and more diverse datasets, together with clinical, imaging, and functional information, will be essential to fully exploit the potential of integromics for biomarker discovery, patient stratification, and personalized therapeutic strategies.

## 3. Emerging Advanced Detection Technologies/Advanced Sensing Technologies

### 3.1. Multiarray System

The advent of multiarray systems (also referred to as multiplex or multi-analyte arrays) is proving to be a significant advancement in the research of IS biomarkers and mechanistic pathways. These platforms allow for the simultaneous measurement of hundreds of analytes (proteins, lipids, cytokines, and metabolites) from a small volume of a biological sample, which is especially important in acute stroke, where sample volume is limited and rapid profiling is a necessity.

Despite promising findings, no molecular panel investigated by means of multiarray systems has provided sufficient accuracy either in differentiating IS from mimics and controls or in stroke subtyping and outcome prediction.

Multiarray systems may also contribute to stroke prognostication.

A large multicenter study measuring 14 plasma biomarkers demonstrated that multi-analyte panels significantly improved the prediction of functional outcomes and recurrent vascular events when combined with clinical information, outperforming individual biomarkers alone [110].

A 92-protein panel was strongly associated with long-term cognitive decline after IS, emphasizing the need to incorporate diverse mechanisms (neuroinflammation, synaptic dysfunction, and neuronal loss) into prognostic models [111].

A comprehensive review of immune–inflammatory biomarkers concluded that composite and multiplex approaches, integrating cytokines and endothelial activation markers, have better prognostic accuracy for infarct expansion, early neurological worsening, poor functional outcome and mortality than any single marker [112].

Multiplexing is expanding beyond plasma: panels of extracellular-vesicle-associated molecules (e.g., neurovascular and inflammatory markers) can stratify patients regarding rehabilitation responsiveness and recovery potential, opening a new avenue for subacute-phase prognostication where conventional biomarkers plateau [113].

There are some important limitations to the clinical use of multiplex arrays. They are non-transportable laboratory-based technologies that are time consuming (sample processing and analysis) and that are not adequate when time is brain.

#### Preclinical Studies

In preclinical stroke research multiarray systems have emerged as powerful tools for the comprehensive profiling of biomarkers in animal models. Martinez-Sanchez et al. found significant differences in biomarker profiles between two models (embolic stroke and permanent MCAO) of brain ischemia in rats and between them and stroke patients [114]. By investigating the expression of IL-6, TNF-α, and glutamate, they found that the embolic stroke model exhibited larger infarct volumes, an IL-6 temporal profile more closely resembling the one observed in humans, and stronger correlations among the three biomarkers, cell death, and infarct size, compared with the permanent MCAO model. More recently, Conti et al. [115] employed a multiplex system for monitoring circulating biomarker levels of cytokines/chemokines at three different time points (pre-stroke, basal, and 24 h after stroke) in stroke mice with and without recanalization. The analysis performed showed that MCA photothrombotic occlusion triggered a strong inflammatory response that is dramatically reduced after recanalization of the vessel.

### 3.2. Single-Molecule Assays

Single-molecule array (SIMOA), combining single-molecule immunocapture with fluorescence detection, can detect extremely low concentrations of biomarkers (at the femtomolar/attomolar level) that were previously not detectable. Moreover, some SIMOA technologies have been recently adapted for high-throughput and multiplex detection [116]. SIMOA represents a breakthrough in biomarker detection. It has been used to detect specific markers of neuronal injury (i.e., GFAP, Neurofilament Light chain [NfL], and S100B) that are released immediately on brain ischemic insult and are therefore possible candidates as diagnostic biomarkers (IS versus hemorrhagic stroke [117] or mimics, in addition to the Prehospital Stroke Scale [118]). SIMOA may become a crucial technology in the identification of stroke prognosis markers. Simoa assays for NfL predict: the final infarct volume [119,120,121]; IS in AF patients [122,123]; early neurological deterioration in minor-stroke patients [124]; short- and long-term poor functional and neurological outcomes (even after adjustment for age, stroke severity, and timing of sampling [119]); as well as white matter hyperintensity progression at 7-year follow-up [119]. These findings must be considered with caution because Nfl is not stroke-specific, and its levels are elevated in any condition involving axonal injury [125].

The clinical question of whether Nfl can significantly contribute to prognostic assessment by increasing the predictivity of imaging has not yet been definitively answered. In any case, given the very low levels of Nfl in peripheral blood (subfemtomolar), SIMOA remains the only appropriate detection technology currently available [126].

SIMOA also has the same limitations as multiarray systems when considering its introduction into acute stroke clinical practice (see above).

#### Preclinical Research

In a recent study, Becktel et al. [127] exploited SIMOA to address the expression of NfL over time in mice after stroke (24 h and 1w–7w after injury), finding that NfL expression was significantly elevated compared to healthy animals throughout the study. In addition, they found a positive correlation between NfL plasma levels and infarct volumes 24 h after injury.

Recently, advances in biosensor technology have enabled rapid and accurate quantification of potential stroke biomarkers. Chen et al. developed and characterized a novel biosensor for quantifying the potential stroke biomarker Neuron-Specific Enolase (NSE) in a mouse model of IS [128]. The proposed biosensor compared to the standard method of analysis has revealed good accuracy. Interestingly, the high sensitivity of the biosensor requires a very small quantity of whole blood (20 μL), and detection is completed within 5 min, which is an advantage for point-of-care (POC) applicability.

### 3.3. Nanoparticle Biosensors

Although the above-mentioned methods for detecting stroke blood biomarkers play a pivotal role in advancing our understanding of stroke pathophysiology and improving patient care, their limitations highlight the need for more rapid, sensitive and accessible diagnostic tools. This gap could be bridged thanks to the emerging field of biosensing. In fact, the advent of nanotechnology-enabled POC biosensors is poised to significantly transform IS diagnosis using rapid, ultra-sensitive, bedside detection of stroke-relevant biomarkers [129,130]. The development of POC biosensors could anticipate stroke diagnosis to pre-hospital settings, guaranteeing faster appropriate treatments, which ultimately lead to better outcomes.

Biosensing technologies are based on the recognition of biological molecules using physical sensors that transform molecules into detectable and quantifiable signals (e.g., optical fluorescence and colorimetric analysis) [123]. Recent reviews highlight that nanomaterial-based sensors can achieve ultra-sensitive detection of stroke-relevant biomarkers at bedside, including low-abundance proteins, microRNAs, and oxidative markers that correlate with brain injury severity and recovery potential. In particular, novel biosensor systems employing nanomaterials can detect biomarkers in whole blood or serum with minimal sample volumes and within minutes [131,132]. For example, an optical biosensor demonstrated sensitive detection of GFAP in human serum, achieving a linear photoluminescence response correlated with biomarker concentration, thus illustrating a potential ambulance-compatible assay for early IS detection [131]. Moreover, research into surface-enhanced Raman spectroscopy (SERS) using silver nanostar conjugates combined with machine learning demonstrated a promising proof of concept for differentiating ischemic versus hemorrhagic stroke in plasma in about 15 min [133]. In addition, the integration of nanotechnology and POC biosensor systems creates a new frontier for IS prognostication, which goes beyond prompt diagnosis with real-time monitoring and outcome prediction. In particular, nanosensor platforms designed for biomarkers such as NSE, S100B, GFAP, and NfL have shown promise in detecting subtle changes that relate to infarct volume, penumbral salvage, and functional outcome trajectories [134].

#### Preclinical Research

Several preclinical studies have applied this technology to investigate the role of different actors of the inflammatory response after IS. In particular, in the last few decades, the role of matrix metalloproteinases (in particular MMP-2 and MMP-9) has been investigated in particular regarding how they exhibit elevated plasma concentrations in patients with IS, suggesting their potential as biomarkers for clinical stroke diagnosis [135,136]. To promote the detection of these enzymes, Gong et al. developed a series of optical interference-free SERS nanotags (CO-nanotags) that allow for multiplexing sensing of different MMPs. This experimental tool has been applied to multiplex detection in a rat model of ischemia, enabling monitoring of IS progression [137]. Given the low invasiveness of the procedure for collecting blood samples from patients, many functionalized nanoparticles and biosensor assays for stroke biomarker detection are directly tested in humans. Preclinical research is strongly focused on testing these particles in other fields, such as diagnostic imaging [138] and treatments [139,140].

## 4. Limitations of Current Preclinical Stroke Models and Translational Challenges

Although preclinical stroke models have been fundamental for elucidating pathophysiological mechanisms and identifying potential therapeutic targets, several limitations should be kept in mind when applying experimental findings to human clinical settings [141,142]. Rodent models, particularly mice, have provided invaluable insights into ischemic injury mechanisms; however, they do not fully represent the complexity of human stroke [142,143]. Biological variables, such as age and sex, and comorbidities, such as hypertension, diabetes, and dyslipidemia, are major determinants of stroke pathophysiology and recovery, but they are frequently underrepresented in animal studies [141,144,145]. In addition, while clinical trial enrollment concerns heterogeneous populations, preclinical investigations are commonly performed in young adult rodents with a homogeneous genetic background and without pre-existing pathological conditions [144,146]. This experimental approach is advantageous because it allows for the investigation of fundamental mechanisms of ischemic injury with minimal potential confounding factors. However, it may limit the translational relevance of findings obtained in these models [141,143].

Translational challenges go beyond demographic and clinical variables: they include additional species-specific differences in vascular anatomy, immune regulation, metabolism, and brain organization [147,148]. In particular, humans and rodents exhibit differences in immune system regulation, metabolism, brain architecture, and regenerative capacity, all of which potentially affect the expression of molecular signatures identified through omics approaches [147]. These limitations are particularly relevant for omics-based studies, since age, sex, metabolic status, and inflammatory state can profoundly shape gene expression, epigenetic regulation, protein abundance, and metabolomic profiles [46,62]. Moreover, variations in experimental protocols, including stroke models (transient vs. permanent occlusion and embolic vs. mechanical models), sample timing, and the type of tissue analyzed, may influence molecular profiles and limit direct comparisons [46,149]. Translational research should integrate findings from animal models, human-derived samples, and large clinical cohorts, ideally combining multi-omics datasets with clinical and imaging parameters to improve the identification of clinically relevant biomarkers [62,141,149].

## 5. Discussion

This review presents the current achievements of the most important emerging technologies related to the field of stroke research that could enhance the background knowledge of stroke physicians. The findings of research using these new technologies will hopefully provide valid contributions for better stroke prevention, diagnosis, treatments, and prognosis. To our knowledge, this is the first comprehensive report of the current status, including possible future scenarios, of clinical and preclinical research in the field of IS.

Heterogeneous pathophysiologies are evidenced, even among patients with the same IS subtype, which highlights the need for personalized approaches based on precision medicine, which in turn is based on biomarker profiling.

Unfortunately, no single-omics technique provides decisive answers. Genomics, in particular GWAS, has identified dozens of possible loci, most of which identify non-coding regions. The role is context-specific, and only an integrated approach between the various omics technologies will be able to provide clinically relevant answers [108,150]. Transcriptomics has provided numerous possible biomarkers, but few studies have been reported, and there are many technical–organizational problems of great relevance. In fact, RNA evaluation has become common practice in some diseases (e.g., cervical cancer, HIV, and breast cancer), but the time it takes to obtain the results varies from 4 to 24 h from blood sample acquisition [46]. This timing is inconceivable in the clinical workflow of acute IS.

Proteomic and metabolomic studies provide promising and potentially useful findings in stroke clinical practice, but they involve significant methodological limitations which lead to inconsistent results. Small sample sizes lead to insufficient statistical power to capture true differences among thousands of measured proteins and metabolites; different patient selection criteria and timings of blood sample acquisition, together with platform heterogeneity, introduce important confounders.

Regarding clinical research, the presence of numerous confounding variables, including age, comorbidities, concomitant therapies, timing of stroke onset, and symptoms, make it extremely difficult to enroll truly homogeneous case series. These difficulties lead to important methodological issues which limit comparison with and reproducibility of omics investigations. One major obstacle, in particular, is the batch effect, which can introduce non-biological variability into omics datasets, thus compromising the reproducibility and comparability of findings across studies. Therefore, standardization of both study samples and procedures, together with rigorous quality control strategies are essential to improve data reliability and allow for metanalyses, facilitating independent validation [151]. Differences in patient selection, stroke subtype, disease severity, timing of sampling, treatment received, and analytical workflows may contribute to inconsistent results across studies. Large multicenter studies using pre-determined candidate biomarkers are more valid and clinically significant than cohort-specific associations.

Furthermore, most omics-based discoveries, such as those reported in this review, currently identify correlations rather than causal relationships. A molecular alteration may reflect a downstream consequence of ischemic injury, an adaptive response, or a true driver of disease progression. Therefore, functional validation approaches are necessary to establish biological relevance and causality. Candidate biomarkers should be validated through complementary strategies, including in vitro experiments using relevant cellular models, genetic manipulation approaches (e.g., knockdown or overexpression studies), animal models, and integration with functional datasets such as single-cell transcriptomics, spatial omics, or proteomic analyses. Combining discovery-based omics approaches with mechanistic validation will be essential to move from biomarker identification toward clinically actionable targets in IS.

Table 2 presents the limitations and possible improvements of the individual omics technologies.

For example, genomics rely on natural genetic variations across populations: most GWAS have been carried out on individuals of European ancestry, thus precluding large applicability to other populations. Nevertheless, a recent study on both European and Chinese populations showed that an integrative GRS may be useful for stroke prognostication [153]. Most limiting factors highlight the need for more standardized protocols, larger and more diverse cohorts, and multicenter validation strategies that render biomarkers reliable tools that can be effectively integrated into the diagnostic workup of IS patients.

Preclinical research provides a crucial contribution: it relies on precise scientific rigor to synchronize biological factors (sex and age) and test time points and comorbidities, making great efforts to develop animal models that better resemble the multifaceted scenario of stroke patients [154,155], using aging animals [156,157,158] and genetically modified animal lines [159,160,161,162]. The development of a murine stroke model that is capable of reproducing human large-vessel occlusion is of fundamental importance in translational research. It allows for the generation of preclinical datasets, which are useful for both studying the mechanisms underlying BBB structural modifications [163] and analyzing post-stroke alterations in functional connectivity [164,165,166,167]. Unfortunately, it is not always possible to translate the same protocols and procedures from preclinical settings to clinical trials. It is often difficult to find the same kits used in the clinical setting, and it is not always possible to repeat mouse serum tests due to the limited amount of available blood from these animals. Conti et al. [115] recently published a paper regarding the investigation of stroke inflammatory biomarkers in mice and humans. The same laboratory was used for both mouse and patient samples, ensuring high reliability of the findings.

It is commonplace in preclinical stroke research to analyze ex vivo brain tissue. Recent rodent studies focus on biomarkers in the penumbral tissue, the battleground where prognosis and final outcome of stroke are decided [45,168]. Brain biopsy tissue analysis is not done in stroke clinical practice or research. Rapid diagnosis is always essential and is based on neuroimaging rather than invasive tissue sampling. This means that peripheral blood and other clinically accessible body fluids are the only viable sources for biomarker assessment in human studies. Use of post-mortem samples is often useless and, in most cases, illegal. In any case the findings can provide definitive information only regarding the type of stroke and its extension and cannot be employed in biomarker studies, given the variability in technical timing of analyses in which it can be performed.

Post-mortem examination, according to the literature, is only useful in cases of uncommon types of stroke because in vivo neuroimaging is almost always reliable [169].

The possibility of analyzing infarcted brain tissue is fundamental for precise identification of the numerous and still undefined aspects of stroke progression. Currently, only preclinical research can furnish information on this topic, because it can use both blood and brain tissue in rapid and experimentally defined times, thus ensuring reproducibility.

Returning to human studies, high-throughput molecular technologies have emerged as a powerful strategy for capturing the systemic signatures of stroke-related biological processes and generating biomarker candidates that may overcome the limitations of single-target approaches. The omics approach is data-driven and therefore unbiased because it yields results that are not based on predetermined hypotheses. Omics generate large-scale molecular datasets that can be integrated with each other (integrated omics, integromics), thereby enabling the identification of complex biomarker signatures as well as individual candidate molecules. Nevertheless, despite years of omics-based research, to date, no biomarker has been applied in stroke clinical practice, due to methodological problems related to the technologies and study designs (see Table 2). The study-design problems depend mostly on the high variability of biological fluids, measurement timing and method, etc. [46]. The different assessment techniques introduce significant inhomogeneities: NMR is commonly used for untargeted metabolomic profiling, whereas LC–MS is mainly used to analyze and quantify specific molecules. As a result, meta-analysis across studies is often difficult, limiting the strength of the available evidence and the possibility of subgroup analyses.

Current technologies, e.g., ELISA, qPCR, and LC–MS/MS, are widely used in research but carry applicative problems regarding complexity, lengthy processing times, and complicated equipment requirements, which do not permit clinical use in the IS time-sensitive condition. In recent years ultra-sensitive detection technologies, such as Simoa and biosensors, have achieved significant breakthroughs, with progressively lower detection limits [123].

The advantages of these nanotechnology-driven platforms in terms of miniaturization, rapid detection, multiplexing, and high sensitivity are promising features for their use in IS biomarker identification. The development of portable devices for using these technologies will allow for their clinical application in the pre-hospital phase and stroke emergency department. Moreover, use of artificial intelligence (AI) and machine-learning (ML) algorithms may boost (i the identification of accurate and specific IS biomarkers that span molecular levels (e.g., transcript → protein → metabolite) and their integration into predictive models; (ii the development of diagnostic and prognostic models that incorporate molecular signatures with clinical and imaging data, potentially enhancing outcome prediction and treatment stratification; and (iii mechanistic network discovery that reveals how molecular interactions contribute to infarct expansion, neuroinflammation and repair, guiding therapeutic target identification. Omics datasets are large, complex, and without a linear relationship. Specific ML modalities are required to reduce dimensionality (e.g., Principal Component Analysis and Uniform Manifold Approximation and Projection) and to screen for the most impactful biomarkers using distinctive algorithms in an attempt to obtain reliable stroke subtyping for use in clinical practice. Computational mapping is also available to translate omics data and investigate the interplay across multiple omics layers (e.g., Graph Convolutional Networks and Multi-Omics Transformation). Among many others, Random Forest, LASSO (Least Absolute Shrinkage and Selection Operator) and XGBoost can create predictive models by selecting the most relevant biomarkers avoiding overfitting. In a recent paper the authors used ML to select the most promising biomarkers related to neurotrophic factors in IS from two large publicly available datasets. The LASSO regression algorithm identified seven genes as potential diagnostic biomarkers for IS [170]. GSE16561 and GSE58294 were analyzed in this study and were downloaded from the GEO database (https://www.ncbi.nlm.nih.gov/geo accessed on 15 April 2025). A total of 2040 neurotrophic factor-related genes (NFRGs) were collected from the GeneCards database.

A recent review on this topic underscored that in spite of their apparent discriminative capabilities, current ML stroke models are inadequate. Larger and better designed studies are necessary to develop robust tools for earlier diagnosis and tailored treatments [109]. Moving forward, the synergy of AI and multi-omics holds strong potential for the enhancement of precision medicine in IS by enabling molecularly informed risk stratification, individualized therapy, and improved prognostic accuracy across diverse patient populations.

Omics sciences have significantly expanded our knowledge of disease biology, yet they have important limitations in identifying reliable prognostic biomarkers. The large magnitude of omics data, where thousands of variables are measured in relatively small patient cohorts, increases the risk of overfitting (when variables outnumber observations) and false-positive findings. Biological variability, technical heterogeneity, batch effects (possible measurement bias), and differences in sample processing further complicate reproducibility across studies and populations. Moreover, many identified molecular signatures lack functional validation and may reflect correlation rather than causation, because most of the studies are cross-sectional.

At present the omics sciences cannot be directly applied in the clinical management of IS patients, because the complex methods used require time, expertise, and advanced technical skills. Omics sciences can be used to identify molecular signatures that assist in the stratification of diagnoses and outcomes, adding information to the standard clinical variables. The potential biomarkers could be determined using simpler technologies, such as POCTs, once they are preclinically and clinically validated.

The aim of this comprehensive review is to highlight the value of the emerging assessment technologies rather than emphasize the identification of the molecules involved in IS.

## 6. Conclusions and Future Directions

In conclusion, all the emerging omics discussed in this work will provide findings that could be translated into clinically operating tools for stroke management in the near future. Obviously, they must not and cannot work alone but in close collaboration with clinics and neuroimaging, supporting clinical decision-making and helping to optimize treatments. By merging clinical and molecular data, accurate predictive models can be built [66], paving the way to personalized treatments and precision stroke medicine.

## Figures and Tables

**Table 2:** Limitations and possible improvement strategies by technology breakdown.

Technology	Limitations	Possible Improvements
**Genomics**	Limited geographical and ethnic representativeness of the samples analyzed in most studies, which further increases the variability of results and reduces their generalizability.	Larger and diverse cohorts studied by means of standardized protocols. Parallel preclinical studies.
**Transcriptomics**	Only a limited number of ncRNAs * have been explored.	Expand the number of ncRNAs * to be studied, by using single-cell and spatial transcriptomics and integrating data with corresponding proteomics [34].
**Proteomics**	Studies found downregulation of synaptic protein and upregulation of inflammatory and coagulation proteins, but with inconsistent results [39,114], possibly due to nonuniformity in study design, biological fluids investigated, timing and detection techniques. Furthermore, large datasets can be difficult to interpret.	Preclinical studies both on fluids and brain tissue may suggest and reinforce the actual biological relevance of specific proteins. Artificial intelligence may support the management and the interpretation of large datasets.
**Metabolomics/Lipoproteomics**	Inconsistent results, possibly due to small sample sizes that preclude stratification for relevant clinical variables, as well as for different timings of blood sampling and detection techniques across studies [152]. Furthermore, large datasets can be difficult to interpret.	Large studies with standardized procedures, possibly including parallel preclinical investigation. Preclinical studies, both on fluids and brain tissue, may suggest and reinforce the actual biological relevance of specific molecules. Artificial intelligence may support the management and the interpretation of large datasets.
SIMOA **	High cost and need for specialized equipment and variability in assay timing, heterogeneity in cohorts and stroke subtypes, and lack of standardized cut-off values.	Large multicenter studies using standardized procedures and inclusion/exclusion criteria to validate cut-off values.
**Multiarray systems**	Still limited by heterogeneity in biomarker selection, variability in sample timing, and the need for large-scale validation complexity, lengthy processing times, and high equipment requirements.	Previous identification of accurate ischemic stroke biomarkers to develop specific multi-analyte arrays.
**Nanoparticle biosensors**	Still confined in research investigations. Sensitive to environmental interference, and complex reading technology.	Previous identification of accurate ischemic stroke biomarkers to develop specific biosensor platforms and standardized procedures.

* Non-coding RNA; ** Single-Molecule Assays.

## Data Availability

No new data were created or analyzed in this study.
